# Mystery or method? Evaluating claims of increased energy expenditure during a ketogenic diet

**DOI:** 10.1371/journal.pone.0225944

**Published:** 2019-12-09

**Authors:** Kevin D. Hall

**Affiliations:** Laboratory of Biological Modeling, National Institute of Diabetes and Digestive and Kidney Diseases, Bethesda, Maryland, United States of America; University of Alabama at Birmingham, UNITED STATES

## Background

The carbohydrate-insulin model of obesity postulates that diets high in carbohydrate result in elevated insulin secretion thereby trapping fat inside adipocytes, decreasing the availability of circulating fuels, and generating a state of “internal starvation” in non-adipose tissues that signals to the brain to suppress energy expenditure and increase hunger [1, 2]. Therefore, isocaloric replacement of dietary carbohydrates with dietary fat should result in decreased insulin secretion, increased circulating fuels, and increased energy expenditure if the carbohydrate-insulin model is valid.

To investigate this possibility, we recently conducted a metabolic ward study of a relatively homogeneous group of 17 men with overweight or class 1 obesity to measure changes in daily energy expenditure as subjects transitioned from four weeks of consuming a high-carbohydrate, high-sugar, baseline diet (BD) to an isocaloric very low-carbohydrate, ketogenic diet (KD) with equal protein for a subsequent four-week period [3]. Subjects spent two consecutive days each week residing in respiratory chambers and the pre-specified primary aim of the study was to measure differences in daily energy expenditure on the final pair of chamber days during each diet period. The study was powered to detect a 150 kcal/d difference in this primary outcome which was pre-specified in the pre-registered clinical study protocol as the minimum effect size that would be deemed physiologically significant [3].

We found no significant difference in daily energy expenditure between the diet periods, with (mean ± SD) 2650 ± 356 kcal/d during the KD versus 2617 ± 395 kcal/d during the BD (p = 0.21) as measured on the final pair of chamber days on each diet in all 17 subjects [4]. Nevertheless, using a repeated measures analysis of all chamber data during the isocaloric diets resulted in a significant increase in chamber energy expenditure that appeared to transiently increase by ~100 kcal/d upon induction of the KD followed by a subsequent waning over time despite rapid and substantial changes in daily insulin secretion, ketosis, and respiratory quotient (RQ) that stabilized within the first week of the KD and persisted throughout the study [5].

The subjects consumed, on average, slightly more energy than they expended during the respiratory chamber days and they completed 90 minutes of mandatory daily exercise at a fixed wattage on cycle ergometers in an attempt to stabilize physical activity expenditure between chamber and non-chamber days. However, on average, the subjects lost body weight and body fat suggesting that non-chamber energy expenditure was greater than the energy expended inside the respiratory chambers. This inference was supported by actigraphy data indicating increased non-chamber physical activity [5].

## Doubly labelled water measurements

The doubly labeled water (DLW) method provides an indirect estimate of the daily average rate of daily CO_2_ production. Calculating energy expenditure requires an estimate of the corresponding daily average RQ which is the ratio of the CO_2_ produced to O_2_ consumed. RQ is strongly influenced by diet composition, level of physical activity, and the overall state of energy balance [6].

We originally reported exploratory DLW measurements that indicated a significant ~150 kcal/d increase in total energy expenditure during the KD as compared to the BD [5]. However, our original DLW calculations used the RQ values measured during the chamber stays which did not account for the differences in energy balance between non-chamber and chamber days. Recently, we published equations to appropriately adjust the RQ values to be used in DLW calculations to account for these differences in energy balance [7]. We found that the revised DLW calculations using the adjusted RQ values resulted in no statistically significant differences in daily energy expenditure during the KD versus the BD periods and these updated DLW results were more consistent with other measures [7].

## Secondary analysis by Friedman and Appel

A secondary analysis of our study was the subject of a new publication in *PLoS ONE* [8] by Mark Friedman and Scott Appel focusing on differences in non-chamber energy expenditure between the diet periods. Non-chamber energy expenditure was determined using the originally reported DLW expenditure values after accounting for the energy expended during the days spent in respiratory chambers [5]. Friedman and Appel reported an apparent ~200–300 kcal/d increase in non-chamber expenditure during the KD as compared to the BD despite no corroborating increases in non-chamber physical activity as measured by actigraphy.

Unfortunately, when Friedman and Appel examined differences between non-chamber and chamber days they failed to adjust the DLW calculations to account for the RQ differences during the non-chamber days as fully described in our recent publication [7]. Rather, Friedman and Appel referred to these well-known effects of energy imbalance on RQ as “hypothetical” and their neglect to appropriately adjust RQ led them to overestimate the differences between non-chamber energy expenditure during the KD versus BD periods.

Indeed, appropriate energy imbalance adjustments of non-chamber RQ resulted in no statistically significant differences in non-chamber energy expenditure between the diet periods, with values of 3395 ± 766 kcal/d during the KD versus 3209 ± 642 kcal/d during the BD (p = 0.15) using data from all 17 subjects (see Supporting Information). Nevertheless, despite the lack of statistical significance, a nominal 185± 508 kcal/d increase in non-chamber energy expenditure during the KD calculated using the appropriately adjusted RQ values could be physiologically important. But to what extent is this apparent difference driven by DLW outliers whose data were incompatible with the physical law of energy conservation?

## Outlier detection by quantifying energy discrepancies

We previously identified two DLW outliers, subjects 04–006 and 04–012, by demonstrating that these subjects exhibited slight gains in weight or body fat despite large apparent negative energy imbalances during the KD as measured by differences between energy intake and RQ-adjusted DLW expenditure of -1741 kcal/d and -821 kcal/d, respectively [7]. The very high DLW expenditure values in these subjects were not corroborated by similarly high expenditure values measured by respiratory chamber or high physical activity measurements via actigraphy [7]. Removing these two outliers reduced the magnitude of the increase in non-chamber energy expenditure between KD and BD periods to only 59± 354 kcal/d (p = 0.53) when the DLW measurements were adjusted for the non-chamber RQ [7].

Friedman and Appel criticized our DLW outlier identification procedure as being post-hoc and noted that the body weight and composition measurements supporting our analyses of the two outlier subjects were conducted on the dates of the dual-energy X-ray absorptiometry (DXA) procedure that deviated by one or two days from the beginning and end of the 14-day DLW measurement periods [8]. Friedman and Appel noted that the pair of daily body weight measurements at the beginning and end of the DLW period in these two outlier subjects indicated slight weight losses rather than the weight gains we reported between the DXA measurements. But these apparent weight losses were merely fluctuations (likely due to body fluid shifts) and did not represent the overall trend determined using all daily body weight measurements during the DLW period. Therefore, we calculated the best-fit linear slopes of daily body weight versus time during the DLW periods which resulted in positive values for both of the outlier subjects indicating states of slight overall weight gain during the KD period as we originally reported.

Friedman and Appel noted that five other subjects had weight changes whose direction appeared to be opposite to that expected from the calculated energy imbalance between intake and expenditure calculated using DLW data unadjusted for non-chamber RQ [8]. However, the average magnitude of energy imbalance in these subjects was less than 17% of the average energy imbalance of the two outliers we previously identified [7] and likely within the precision of the individual measurements. For example, the precision of the DLW method is ~ 8–15% [9]

We recently published a method for systematically quantifying the amount of unaccounted energy in individual subjects to detect and eliminate outliers whose data are incompatible with the physical law of energy conservation [10]. Our method requires estimating each subject’s rate of change in stored body energy which was calculated as the best fit linear rate of weight change (in kg/d) from the daily body weight measurements during the DLW period multiplied by the energy density of 5725 kcal/kg calculated from the DXA measurements during the final 2 weeks of the KD. The energy discrepancy of each subject was then calculated as energy intake minus DLW expenditure (adjusted for non-chamber RQ) minus the rate of change in body energy stores. Detailed calculations are provided in the Supporting Information.

**Fig 1:**
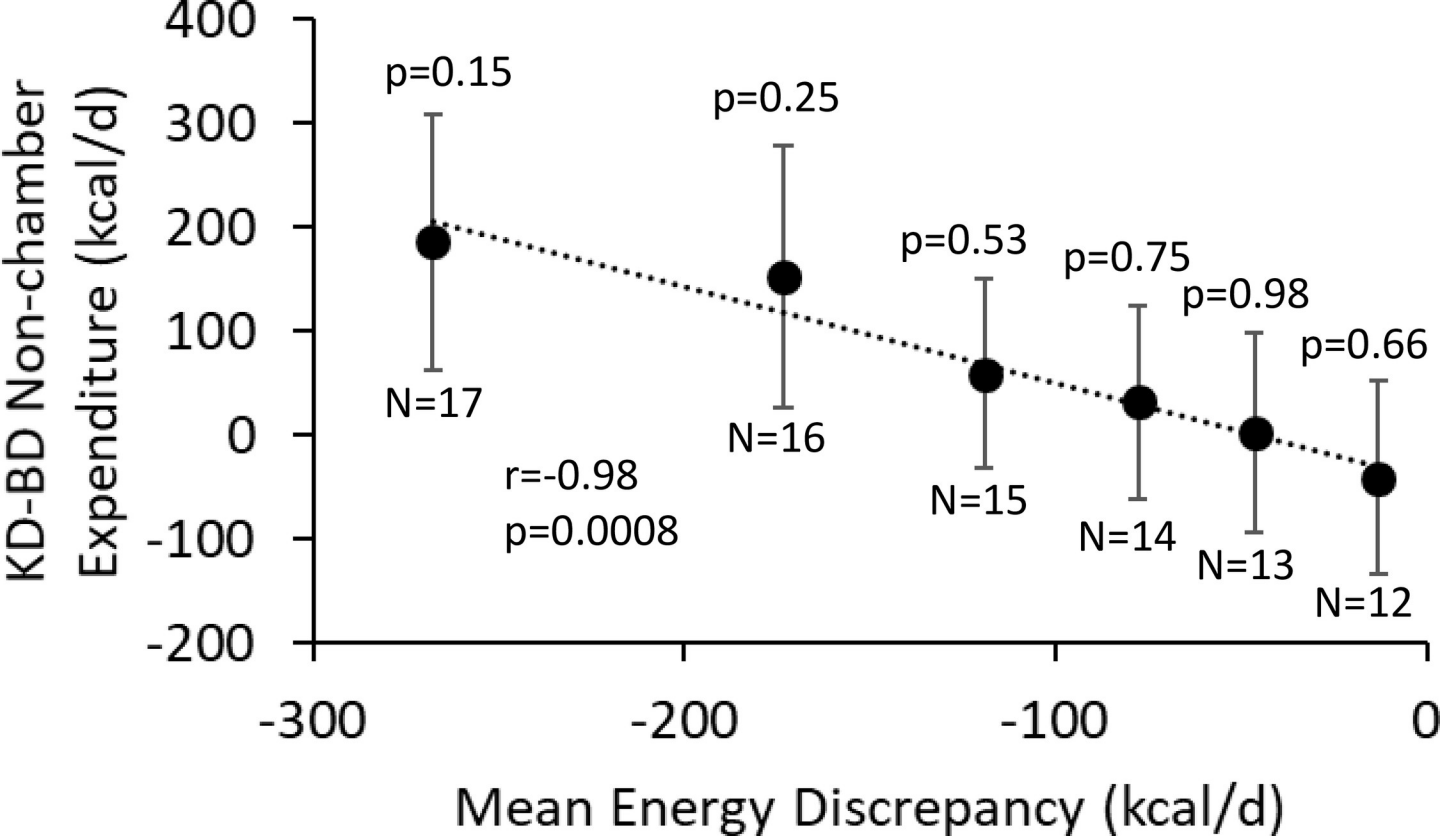
Outlier subjects were responsible for the potentially meaningful differences non-chamber energy expenditure between diet periods. Differences in non-chamber energy expenditure (adjusted for non-chamber RQ) between the isocaloric ketogenic diet (KD) and baseline diet (BD) periods as a function of the mean energy discrepancy as subjects were sequentially removed based on the magnitude of their energy discrepancy. The magnitude of the nominal differences in non-chamber energy expenditure declined as subjects with the largest magnitude energy discrepancies were removed. Mean ± SEM.

Fig 1 plots the mean non-chamber energy expenditure differences between KD and BD periods (adjusted for non-chamber RQ) as a function of the mean energy discrepancy during the KD as subjects were sequentially removed based on the magnitude of their energy discrepancy. The left-most data point in Fig 1 includes all 17 subjects and the mean energy discrepancy was about -270 kcal/d. The original two outliers, subjects 04–006 and 04–012, had the largest magnitude energy discrepancies of -1778 kcal/d and -984 kcal/d, respectively. Sequentially removing subjects with the greatest magnitudes of energy discrepancy during the KD resulted in a progressive decline in the mean difference in non-chamber energy expenditure between diet periods (r = -0.98; p = 0.0008). (Note some energy discrepancies in individual subject data are expected based on the limited precision of the measurements.) Therefore, the potentially physiologically important differences in non-chamber energy expenditure between diet periods were driven by outliers whose data were incommensurate with the law of energy conservation. Furthermore, non-chamber energy expenditure differences between KD and BD periods were not statistically significant independent of the number of outliers sequentially removed based on their magnitude of energy discrepancy.

## Conclusion

Friedman and Appel’s secondary analysis failed to account for RQ differences between chamber and non-chamber days and therefore resulted in the erroneous conclusion that non-chamber energy expenditure was significantly increased during the KD period. Methods to appropriately adjust the RQ values used in DLW calculations were previously published [7] and implementing those adjustments resulted in no significant differences in non-chamber energy expenditure between the diet periods. After removing two clear DLW outliers whose data violated the physical law of energy conservation, the difference in non-chamber energy expenditure between the diet periods was quantitatively small (<60 kcal/d) and not statistically significant. Furthermore, the DLW energy expenditure values adjusted for non-chamber RQ were in agreement with measurements of body weight, composition, physical activity, and respiratory chamber expenditure.

In contrast, Friedman and Appel’s DLW calculations that failed to appropriately account for non-chamber RQ resulted in apparent increases in non-chamber expenditure during the KD that were uncorroborated by other measurements. The mechanisms proposed by Friedman and Appel for such apparent non-chamber energy expenditure differences between diet periods are somewhat mysterious. For example, non-chamber muscular work efficiency was suggested to have decreased during the KD period thereby resulting in increased non-chamber physical activity expenditure, but this hypothesis is not supported by the similar efficiency of fixed-wattage cycling exercise between the diet periods [5] and actigraphy measurements indicated either a decrease or no significant differences in non-chamber physical activity between KD and BD periods [8]. Friedman and Appel also suggested that the energy-requiring process of gluconeogenesis may have been responsible for increased non-chamber expenditure during the KD. However, it is unclear why non-chamber days would require additional gluconeogenesis as compared to chamber days. Furthermore, any such increase in non-chamber gluconeogenesis could not possibly amount to the ~120–180 g/d calculated to be required to explain Friedman and Appel’s reported ~200–300 kcal/d differences [11] in non-chamber energy expenditure during the KD as compared to the BD periods.

In conclusion, the mysterious apparent increase in non-chamber expenditure that Friedman and Appel claim supports the carbohydrate-insulin model is more likely explained by methodological flaws in their DLW calculations that resulted in overestimating the difference in non-chamber expenditure between KD and BD periods.

## Supporting information

S1 File Hall PLoS ONE 2019 data. (XLSX)

## References

[1] Ludwig DS, Ebbeling CB. The Carbohydrate-Insulin Model of Obesity: Beyond "Calories In, Calories Out". JAMA internal medicine. 2018;178(8):1098–103. Epub 2018/07/05. 10.1001/jamainternmed.2018.2933 29971406 PMC6082688

[2] Ludwig DS, Friedman MI. Increasing adiposity: consequence or cause of overeating? JAMA. 2014;311(21):2167–8. 10.1001/jama.2014.4133 . 24839118

[3] Hall KD. Effect of a Eucaloric Ketogenic Diet on Energy Expenditure: A Pilot Study / NIDDK Clinical Protocol: Open Science Framework; 2014 [cited 2019 9/16/2019]. Available from: https://osf.io/fj2xm/.

[4] Hall KD, Chen KY, Guo J, Leibel RL, Mayer LE, Reitman ML, et al Reply to DS Ludwig and CB Ebbeling. Am J Clin Nutr. 2016;104(5):1488–90. Epub 2016/11/03. 10.3945/ajcn.116.143628 27802997 PMC5081725

[5] Hall KD, Chen KY, Guo J, Lam YY, Leibel RL, Mayer LE, et al Energy expenditure and body composition changes after an isocaloric ketogenic diet in overweight and obese men. Am J Clin Nutr. 2016;104(2):324–33. 10.3945/ajcn.116.133561 . 27385608 PMC4962163

[6] Elia M. Energy equivalents of CO2 and their importance in assessing energy expenditure when using tracer techniques. Am J Physiol. 1991;260(1 Pt 1):E75–88. Epub 1991/01/01. 10.1152/ajpendo.1991.260.1.E75 . 1899005

[7] Hall KD, Guo J, Chen KY, Leibel RL, Reitman ML, Rosenbaum M, et al Methodologic considerations for measuring energy expenditure differences between diets varying in carbohydrate using the doubly labeled water method. Am J Clin Nutr. 2019 Epub 2019/04/28. 10.1093/ajcn/nqy390 . 31028699 PMC6499509

[8] Friedman MI, Appel BE. Energy expenditure and body composition changes after an isocaloric ketogenic diet in overweight and obese men: a secondary analysis of energy expenditure and physical activity. PLoS ONE. 2019:e222971. 10.1371/journal.pone.0222971 PMC6901216 31815933

[9] Black AE, Cole TJ. Within- and between-subject variation in energy expenditure measured by the doubly-labelled water technique: implications for validating reported dietary energy intake. Eur J Clin Nutr. 2000;54(5):386–94. 10.1038/sj.ejcn.1600970 . 10822285

[10] Hall KD, Guo J, Speakman JR. Do low-carbohydrate diets increase energy expenditure? Int J Obes (Lond). 2019 Epub 2019/09/25. 10.1038/s41366-019-0456-3 . 31548574 PMC8076039

[11] Veldhorst MA, Westerterp-Plantenga MS, Westerterp KR. Gluconeogenesis and energy expenditure after a high-protein, carbohydrate-free diet. Am J Clin Nutr. 2009;90(3):519–26. 10.3945/ajcn.2009.27834 . 19640952

